# Diagnosis of Appendiceal Neuroendocrine Tumors Following Incidental Appendectomy During Benign Gynecologic Laparoscopic Surgeries: A Case Series

**DOI:** 10.7759/cureus.41135

**Published:** 2023-06-29

**Authors:** Paulina C Altshuler, Peter M Schultze

**Affiliations:** 1 Obstetrics and Gynecology, Intermountain Healthcare, Saint Joseph Hospital, Denver, USA; 2 Obstetrics and Gynecology, Colorado Permanente Medical Group, Aurora, USA

**Keywords:** mature cystic ovarian teratoma, total laparoscopic hysterectomy, laparoscopic right hemicolectomy, laparoscopic surgery for endometriosis, laparoscopic technique, appendiceal endometriosis, appendiceal neuroendocrine tumor, endometriosis and chronic pelvic pain

## Abstract

Gastrointestinal neuroendocrine tumors, although relatively rare, are one of the most common appendiceal neoplasms. Patient symptoms can range from asymptomatic to acute appendicitis, and these tumors are often diagnosed after histopathological evaluation. This case series describes five separate cases of appendiceal neuroendocrine tumors diagnosed by histopathological review following incidental appendectomy during benign gynecologic laparoscopic surgeries at a single multispecialty group. Each case had a preoperative diagnosis of chronic pelvic pain. Intraoperatively, the appendix appeared scarred, adhered, or nodular. Two patients required a right laparoscopic hemicolectomy for the management of the appendiceal neuroendocrine tumor. As a result of these findings, it is recommended that the appendix be routinely evaluated during gynecologic surgeries and, if abnormal in appearance, appendectomy should be performed. Additionally, laparoscopic gynecologic surgeons should receive appendectomy training to aid with the early diagnosis and treatment of appendiceal neuroendocrine tumors.

## Introduction

Gastrointestinal neuroendocrine (carcinoid) tumors are relatively uncommon. However, neuroendocrine tumors are one of the most common neoplasms of the appendix with an incidence ranging from 0.3% to 0.9% [1]. Appendiceal neuroendocrine tumors (ANET) are most often diagnosed between forty and fifty years old, are more common amongst females, and can present as acute appendicitis or by incidental diagnosis following histopathological evaluation [2, 3].

This case series describes five separate cases of appendiceal neuroendocrine tumors diagnosed by histopathological review following incidental appendectomy during benign gynecologic laparoscopic surgeries. This retrospective chart review was conducted between January 2011 and January 2022 at a large multispecialty group (averaging 600 minimally invasive hysterectomies annually) with four minimally invasive gynecologic surgeons (MIGS) certified to perform appendectomies. This article was previously presented as a meeting abstract at the Society for Gynecologic Surgeons Annual Scientific Meeting in Tucson, AZ from March 19-22, 2023.

## Case presentation

Case 1

A 32-year-old nulliparous Caucasian female with chronic pelvic pain (CPP) and endometriosis was managed by benign gynecology for over ten years for dysmenorrhea, bowel and bladder discomfort, and dull pelvic pain. She had a history of sexual abuse, post-traumatic stress disorder (PTSD), and asthma. For her CPP, she tried non-steroidal anti-inflammatory drugs (NSAIDs), oral contraceptive pills (OCPs), an etonogestrel/ethinyl estradiol vaginal ring, oral tetrahydrocannabinol (THC), topical cannabidiol (CBD), physical therapy, hydromorphone, eye movement desensitization and reprocessing (EMDR) trauma therapy, and leuprolide acetate. Preoperative transvaginal ultrasound detailed a normal uterus and ovaries bilaterally, and an abdominal and pelvic computerized tomography (CT) scan showed a normal appendix. She was offered surgical management for her CPP and underwent a total laparoscopic hysterectomy, bilateral salpingectomy, endometriosis excision, and concurrent appendectomy due to an erythematous and irritated scar at the appendiceal base. Intraoperative findings were notable for endometrial implants along the peritoneum and left ovary, nodular scarring in the posterior cul-de-sac, and a globular uterus. Pathology was remarkable for endometriosis and a well-differentiated appendiceal neuroendocrine carcinoma measuring 0.3 centimeters (cm) that invaded the submucosa with negative margins. The ANET was assigned Stage I (pT1) and there was no indication for further appendiceal surveillance. She remained on OCPs for Stage II endometriosis suppression and had no postoperative complications.

Case 2

**Figure 1:**
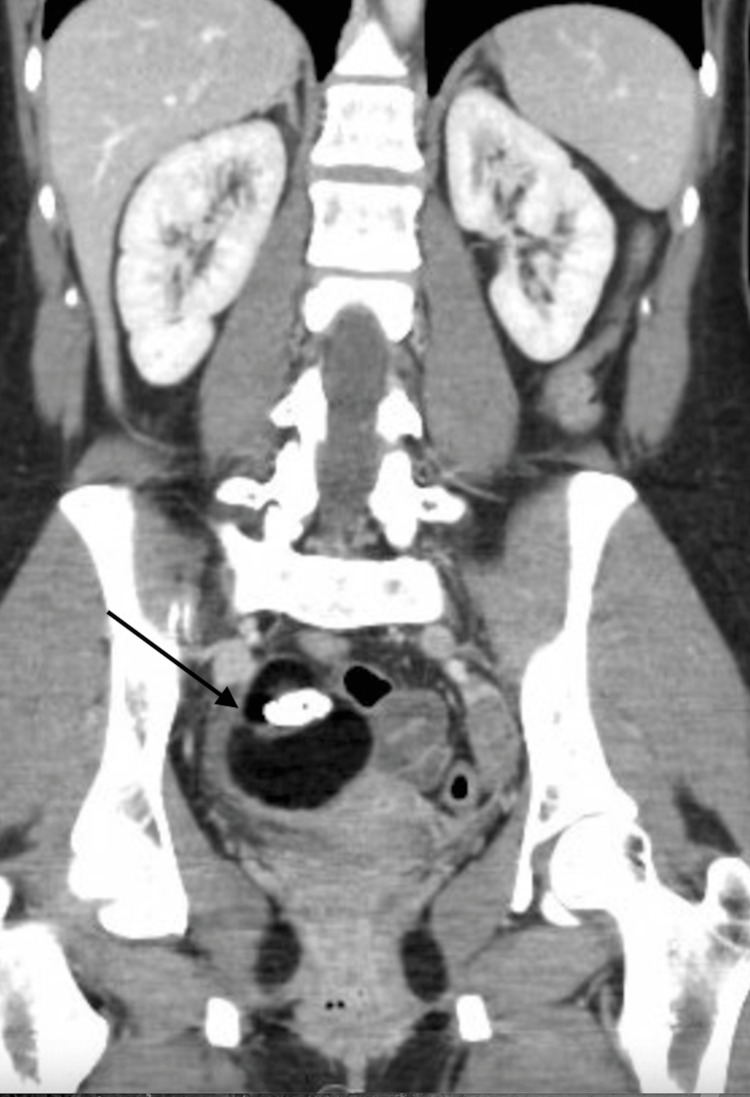
Predominantly fat-containing right ovarian mass with peripheral soft tissue component and central dystrophic calcifications measuring 5.1 x 6.0 x 6.6 centimeters consistent with a cystic teratoma.

A 29-year-old nulliparous Caucasian female with CPP and a suspected right ovarian mature teratoma developed right lower quadrant dull aching and cramping following abdominal trauma at work. She denied dyspareunia and urinary or bowel symptoms. She was previously diagnosed with papillary thyroid cancer and had resultant post-thyroidectomy hypothyroidism. She also used tobacco products daily. She did not attempt medical treatments prior to surgery. A preoperative transvaginal ultrasound and an abdominal and pelvic CT scan demonstrated a six-centimeter circumferential suspected mature teratoma and normal appendix (Figure 1). She proceeded with surgical management via laparoscopic right salpingo-oophorectomy, lysis of adhesions, and incidental laparoscopic appendectomy due to nodularity at the distal tip of the appendix with filmy adhesions to the sidewall. Intraoperative findings were notable for the previously described cyst. Pathology was remarkable for a mature cystic teratoma and a well-differentiated appendiceal neuroendocrine carcinoma measuring 2.2 cm that invaded through the muscularis propria into the mesoappendiceal fibroadipose tissue. The ANET was assigned Stage III (pT3) and she underwent an uncomplicated laparoscopic right hemicolectomy and lymphadenectomy with general surgery eight weeks later. The right colon and terminal ileum were negative for residual malignancy and 19 lymph nodes were negative. She received routine post-operative care and had no complications.

Case 3

A 21-year-old nulliparous Caucasian female was managed by benign gynecology for three years of generalized CPP, right lower quadrant pain, heavy menstrual bleeding, dysmenorrhea, and abdominal bloating. She denied dyspareunia and urinary or bowel symptoms. Her history was remarkable for iron-deficiency anemia, asthma, and low back pain. For the management of her CPP, she had previously tried NSAIDs, OCPs, Naproxen, THC, and pelvic floor physical therapy. Preoperative abdominal and pelvic CT detailed a normal appendix. She underwent an uncomplicated diagnostic laparoscopy, radical peritonectomy, bilateral uterosacral ligament excision, levonorgestrel intrauterine device insertion, and incidental laparoscopic appendectomy due to a scarred appendix. Intraoperative findings included endometrial implants along the peritoneum, right ovary, and uterosacral ligaments consistent with Stage III endometriosis, sigmoid colon adherent to the left pelvic sidewall, and normal-appearing uterus. Pathology was remarkable for endometriosis and a well-differentiated appendiceal neuroendocrine carcinoma measuring 0.4 cm that invaded the muscular wall of the appendix with negative margins. The ANET was assigned Stage I (pT1a) and there was no indication for further appendiceal surveillance. She had an uncomplicated postoperative recovery.

Case 4

**Figure 2:**
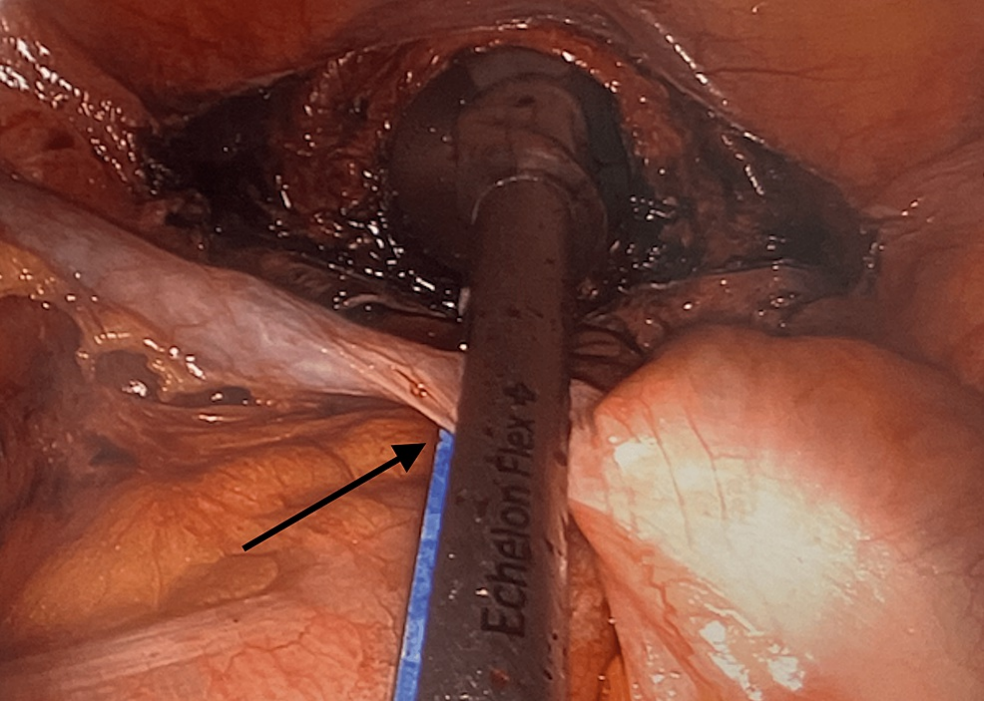
Appendectomy via vaginal advancement of the Endostapler following total laparoscopic hysterectomy.

A 41-year-old Caucasian Gravida 3 Para 2012 (G3P2012) female was managed by benign gynecology for dysmenorrhea, heavy menstrual bleeding despite megestrol and OCPs, and suspected endometriosis. She denied dyspareunia and bowel or bladder symptoms. Her history was remarkable for a major depressive disorder, alcohol dependence, pancreatitis, fatty liver, and a thyroid nodule. Preoperative transvaginal ultrasound showed a retroflexed uterus with normal bilateral ovaries and physiologic free fluid. She underwent an uncomplicated total laparoscopic hysterectomy, bilateral salpingectomy, bilateral ureterolysis, cystoscopy, and incidental laparoscopic appendectomy via the vaginal orifice for a nodular appendix with thickened and indurated areas (Figure 2). Intraoperative findings were notable for endometrial implants along the posterior cul-de-sac and uterosacral ligaments with otherwise normal-appearing reproductive organs consistent with Stage I endometriosis. Pathology was remarkable for endometriosis and extensive adenomyosis along with a well-differentiated appendiceal neuroendocrine carcinoma measuring 0.7 cm that invaded the subserosal tissue and visceral peritoneum with negative margins. The ANET was assigned Stage I (pT1a) and there was no indication for further appendiceal surveillance. Her postoperative course was complicated by poor pain control requiring overnight hospital admission for pain management.

Case 5

A 23-year-old African American G1P1 female was managed by benign gynecology for five years of CPP and dysmenorrhea for which she had tried OCPs. She denied dyspareunia and urinary or bowel symptoms. She also had a history of chlamydia infection, migraines, and major depressive disorder. Preoperative transvaginal ultrasound detailed a normal uterus with a two-centimeter suspected hemorrhagic ovarian cyst. She underwent a diagnostic laparoscopy, excision of endometriosis, lysis of adhesions, and incidental laparoscopic appendectomy for an appendix with endometrial implants and adhesions from the distal tip to the cecal mesentery. Intraoperative findings were notable for endometrial implants along the left anterior broad ligament, uterosacral ligaments, and right pelvic sidewall consistent with Stage II endometriosis, with otherwise normal-appearing reproductive organs. Pathology was remarkable for endometriosis and a well-differentiated appendiceal neuroendocrine carcinoma measuring 1.6 centimeters that invaded the muscularis propria and had an indeterminate proximal margin with possible lymphovascular extension. The tumor was assigned pT1b and staging was inconclusive due to uncertainty regarding lymphovascular spread along the proximal appendiceal margin. As a result, general surgery performed a laparoscopic right hemicolectomy and lymphadenectomy six weeks later. The right colon and terminal ileum were negative for residual malignancy and 45 lymph nodes were negative, which concluded a Stage I ANET. She was placed on leuprolide acetate for endometriosis suppression and had an uncomplicated recovery from both procedures.

## Discussion

During gynecologic procedures for the management of endometriosis or chronic pelvic pain, appendectomies have become more common. Endometriosis implants have been identified on the appendix during a histopathologic review, and studies have suggested that the appendix may contribute to chronic pelvic pain [4,5]. Coexisting endometriosis and appendiceal neuroendocrine tumors have also been identified during gynecologic surgeries [6,7]. One systematic review identified that the rates of ANETs are the same in endometriosis patients as in the general population, which suggests that endometriosis is not a risk factor for ANETs [8]. During laparoscopic gynecologic surgery, the appendix should be routinely and systematically evaluated. If the appendix appears abnormal, appendectomy should be performed for early diagnosis of appendiceal neuroendocrine tumors.

## Conclusions

Each of the five cases presented had a preoperative diagnosis of chronic pelvic pain. Four patients had a postoperative diagnosis of endometriosis after a histopathologic review, and one patient was confirmed to have a mature cystic teratoma. The patients ranged from 21 to 41 years old. Following the staging of the ANETs, two patients required a right hemicolectomy. In each of the five cases, the appendix appeared nodular, scarred, or adhered to adjacent structures. In order to preserve a minimally invasive technique with five-millimeter trocars following total laparoscopic hysterectomy, the endoscopic stapler was deployed via the vaginal orifice, and the appendix was removed vaginally using an endoscopic specimen bag.

Laparoscopic gynecologic surgeons, particularly those routinely managing endometriosis and chronic pelvic pain, should receive appendectomy training to aid with the early diagnosis of appendiceal neuroendocrine tumors. Training for appendectomies and management of associated complications should become a required component in obstetrics and gynecology residency programs to adequately prepare gynecologic surgeons.
